# A Report of Cases Treated with Ichthyolated Emulsion Compound

**Published:** 1908-11

**Authors:** John Roy Williams

**Affiliations:** Greensboro, N. C.


### A REPORT OF CASES TREATED WITH ICHTHYOLATED EMULSION COMPOUND.

BY JOHN ROY WILLIAMS, M. D., GREENSBORO, N. C.

Ichthyol, as a valuable adjunct in the treatment of tuberculosis, has been recognized for over twenty years. It has not been used as largely however, because of its disagreeable odor and taste. Heretofore it has been prescribed in aqueous solution, in capsules or as the gelatin-coated pills.

It is a recognized clinical fact that Ichthyol improves the digestion and assimilation, with little or no irritation of the gastro-intestinal mucous membrane if carefully applied. It lessens tissue destruction, especially the albumens, as shown by deminished nitrogenous excretion; it increases the flesh and strength by increasing the appetite, digestion and assimilation, thereby lessening the outgo and increasing the income. It is eliminated chiefly by the kidneys, in the form of sulphur compounds, acting as a diuretic. It contracts the capilliaries, improving the general circulation tends to rapidly convert a purulent sputum into a mucoid sputum, making it more fluid and easily raised, improving the drainage from the diseased foci and lessening the absorption, which is the chief cause of rapid heart and fever. It often rapidly re-establishes the menstruation in tubercular anaemic women, and the night-sweats in most cases soon stop. It improves the nutrition of the heart throughout its constricting action on the coronary arterioles, and indirectly, through its several physiological actions, lessens blood destruction and assists in building up the same.

1.—Potter says, "the particular value of ichthyol is due to its non-irritant quality and the large proportion of sulphur contained therein. It retards the disintegration of albumens and favors their formation and accumulation."

2.—Bartholow says, "It increases assimilation and hinders retrograde metamorphosis, whereby the nutrition is improved and the body weight is brought up to the normal level. Has a decided antiseptic action and is fatal to pathogenic organisms. It increases the volume and force of the circulation. Has a remarkable power to check waste, the urinary solids and nitrogenous excreta being greatly deminished. Hence under its administration, the body weight increases, the income is promoted and the outgo is lessened, and these important results are accomplished without in any way imparing digestion or irritating the gastro-intestinal mucous membrane."

3.—Cohn says, "it increases the strength of the organism and places itself in a favorable position to carry on a successful warfare against the bacilli of tuberculosis. In advanced cases improvement often follows when Cod Liver Oil and Creosote have failed."

4.—Branthomme says, "he considers the action of ichthyol similar to creosote, but without the disadvantages of the latter.

It is less irritating to the stomach than creosote, deminishes expectoration, causes an increase in weight, improves the general condition of the patient, and restores menstruation in tubercular anaemic women."

5—Combemale and Desoil report that, "during fourteen months all tubercular patients at the Charite Hospital at Lilly were treated with ichthyol. There was prompt improvement in the general health of the patients, as manifested by the disappearance of the night-sweats, gain in weight and strength, and a re-appearance of the menses. The expectoration is almost invariably lessened in amount and made more fluid, so that coughing is easier."

6.—Stubbert reports that, "of all drugs used in the Loomis Sanitarium at Liberty, N. Y., for phythysis, ichthyol had yielded the best results. Under its influence the sputum was more easily brought up, because less yellow and more of a mucoid appearance. There was amelioration of chills, sweats and fever."

7—Williams says, "he has used ichthyol in several hundred cases of pulmonary tuberculosis, with good results in a large majority of cases. He has found the appetite and digestive powers increased, the daily average of temperature lowered and the sweats in most cases to cease. The bacilli rapidly show degenerative changes, with a gradual disappearance of them from the sputum. The cough is modified, the expectoration becoming easier, the sputum becoming more fluid, losing its purulent character, and gradually decreasing in amount."

The odor and taste of ichthyol have for the past twenty years, prevented many consumptives from having the advantages of this remedy. Prescribed in aqueous solution, it is foul tasting and odorous, very often nauseating to a degree to produce vomiting. The very large dose, which sometimes becomes necessary to obtain its benefits, has added to these disagreeable features. Given in capsule, the odor and taste have not always been eliminated; also some patients have found it very disagreeable or impossible to swallow the capsule, due, however, to a neurotic cause, yet nevertheless existant. It was also difficult to get the patients to take enough water with each dose to make a dilute solution in the stomach, so as to prevent irritation, with consequent eructation or "rifting." Since the gelatin-coated pill often passes out with the stool, being undissolved in the stomach or bowel, the therapeutic action of ichthyol in this form can not be depended upon.

For the past several months, I have been using ichthyol in the form of ichthyolated emulsion compound. It contains ichthyol, grs. x, apinol, min. x, ol. sassafras, min. v, ol. olivae, ol. gossypii seminis, glycerinum and mucil, acacae, qs oz. 1. The odor and taste of the ichthyol are disguised. The apinol incorporated, which has all of the physiological actions of creosote, with much less tendency to irritation, enhances the action of the ichthyol and eliminates the necessity of such large doses.

I find that this emulsion gives all the physiological effects of ichthyol and creosote. That it is well borne by the stomach and bowel, has a tendency to overcome constipation, is almost a specific for indicanuria. I give it in a glass of cold milk, six times daily, beginning with a teaspoonful at a dose, slowly increasing until the patient is taking a tablespoonful at each dose.

I have seven cases which I have treated with this emulsion, I wish to report in a general way. Four of them were first stage cases, all having slight fever and accelerated pulse. Three of these cases were complicated with a tendency to constipation. The average length of time treated was nine weeks, and the average gain in flesh was 19 pounds. The highest gain was 26 pounds, the lowest was 12 pounds. All were having a normal pulse and temperature, and the cough and expectoration had disappeared at the expiration of treatment. There seemed to be an arrest or cure of the disease in each instance.

There was one second stage case, a rather large woman, weighing 144 pounds when beginning treatment Her temperature was 101.5, pulse 114. After eleven weeks' treatment she had gained 23 pounds, the temperature and pulse being normal, and the cought and expectoration had almost entirely ceased. She stopped treatment and has been lost sight of. She gave promise of making a complete arrest or cure of the disease.

The remaining two cases, were third stage cases, one complicated with tubercular laryngitis, the other with tubercular enteritis. Both had both upper lobes involved, and were having high temperature and rapid pulse. The cases complicated with tubercular larynigitis, was treated for fourteen weeks, with considerable improvement in the lung condition, no change in the larynx, with prompt reduction in temperature and slower heart action, gained must in strength and added 8 pounds in weight. He stopped treatment, went home, and has since grown worse. The other third stage case, complicated with tuberculosis entertitis, made a far greater improvement. The bowel condition was apparently arrested by the use of magnesium sulphate, in five grain doses every two hours for three weeks. The lung condition greatly cleared, becoming quiescent, and the patient gained 26 pounds in seventeen weeks. Her temperature and pulse reached the normal. She went home, from where she reports she is doing as well as when she left my care.

I employed in all these cases, the usual hygenic and dietectic treatment, and with the two third stage cases, and one of the first stage cases, I employed tuberculin. While I have use ichthyol in capsules in my practice in a large number of cases, the results I have obtained with ichthyol in the form of an emulsion, have been far superior. While ichthyolated emulsion compound has not produced these good results unaided, yet I feel and believe that it has been a most valuable adjunct.

*References.*—

1. Mat. Med., Pharm. and Ther., 1899, P. 488.
2. Mat. Med. and Ther., 1899, P. 349.
3. Inter. Med. Annual, 1898, P. 417.
4. La France Med., Nov. 12, 1897.
5. Med. News, Vol. LXXIII, P. 144.
6. Med. Rec., Vol. LV, P. 730.
7. Charlotte Med. Jour., Vol. XIII, P. 17.

